# Electrochemical Biosensors Employing Natural and Artificial Heme Peroxidases on Semiconductors

**DOI:** 10.3390/s20133692

**Published:** 2020-07-01

**Authors:** Bettina Neumann, Ulla Wollenberger

**Affiliations:** Institute of Biochemistry and Biology, University of Potsdam, 14476 Potsdam, Germany; bettina.neumann@uni-potsdam.de

**Keywords:** electrochemical biosensors, heme, peroxidases, semiconductors, peroxidase mimics

## Abstract

Heme peroxidases are widely used as biological recognition elements in electrochemical biosensors for hydrogen peroxide and phenolic compounds. Various nature-derived and fully synthetic heme peroxidase mimics have been designed and their potential for replacing the natural enzymes in biosensors has been investigated. The use of semiconducting materials as transducers can thereby offer new opportunities with respect to catalyst immobilization, reaction stimulation, or read-out. This review focuses on approaches for the construction of electrochemical biosensors employing natural heme peroxidases as well as various mimics immobilized on semiconducting electrode surfaces. It will outline important advances made so far as well as the novel applications resulting thereof.

## 1. Introduction

Heme peroxidases are popular tools for a variety of bioanalytical techniques where they serve e.g., as reporter enzymes in affinity-based assays or as recognition elements in biosensors [1]. The redox enzymes are especially attractive biocatalysts for the construction of electrochemical biosensors for the detection of peroxides, phenolic compounds, or aromatic amines [2]. Phenols and derivatives are highly abundant toxic wastewater contaminants of e.g., the plastic, paper, and pharmaceutical industries [3,4], while the determination of peroxide concentrations is of high relevance for e.g., the food, pharmaceutical, paper, and textile industries [5,6,7]. As by-product of many oxidases like glucose oxidase (GOx) or cholesterol oxidase, its detection is further important for diagnostics [2]. In addition, the in vivo relevance of hydrogen peroxide as a signaling molecule [8], cell toxin, and disease indicator [9] has gained more attention, invoking the need for highly sensitive biosensors. 

Electrochemical biosensors employing heme peroxidases immobilized on classical electrode materials like carbon or noble metals have been extensively reviewed in the past [2,10,11,12]. Semiconducting materials, on the other hand, have not gained that much attention in this field, although they have been employed already since decades as substrates for the immobilization of enzymes [13,14,15,16,17,18]. The ability of semiconductors to control charge accumulation and release by potential, light or heat cannot only be exploited for photovoltaic systems, solar energy harvesting, and conversion [19,20], but can also open up new possibilities for the application of enzymes like heme peroxidases in photoelectrocatalytic devices [21]. 

Despite their wide use and large potential, the structural properties of heme peroxidases interfere with their production on high scales and, more importantly, can impede their performance in electrochemical biosensors. By the use of chemical and biological engineering techniques and computational methods, researchers have therefore developed various alternative chemical and biological molecules that could serve as mimics for heme peroxidases, catalyzing the same or even more reactions, but outperforming the natural enzymes in terms of electrocatalytic activity or stability under harsh conditions. In combination with the development of new electrode surfaces and immobilization techniques, new efficient biosensors based on peroxidase reactions have been designed.

This review compiles approaches for the development of electrochemical biosensors employing natural heme peroxidases as well as various mimics—ranging from nature-derived heme-peptide complexes to fully synthetic heme derivatives—immobilized on semiconducting electrode surfaces. It will outline important advances made so far as well as the novel applications resulting thereof.

## 2. Heme Peroxidases and Their Mimics

### 2.1. Biochemistry of Heme Peroxidases

Heme peroxidases, EC 1.11.1.7, catalyze the oxidation of a broad variety of reductants (AH_2_) by peroxides, usually hydrogen peroxide, following Equation (1) [22,23]:

H_2_O_2_ + 2AH_2_ → 2H_2_O + 2AH▪. (1)

Heme peroxidases are found in all domains of life. Yet, for bioanalytical applications secretory plant peroxidases, constituting family III of the superfamily of peroxidases-catalases [24], are mostly used. The heme peroxidase from horseradish (HRP) is probably the best-studied member of this family and therefore, the most popular peroxidase for the construction of electrochemical biosensors. However, systems employing other heme peroxidases e.g., from soybean [25,26,27], tobacco [28,29,30], as well as peanut and sweet potato [28,31], have been reported. 

**Figure 1:**
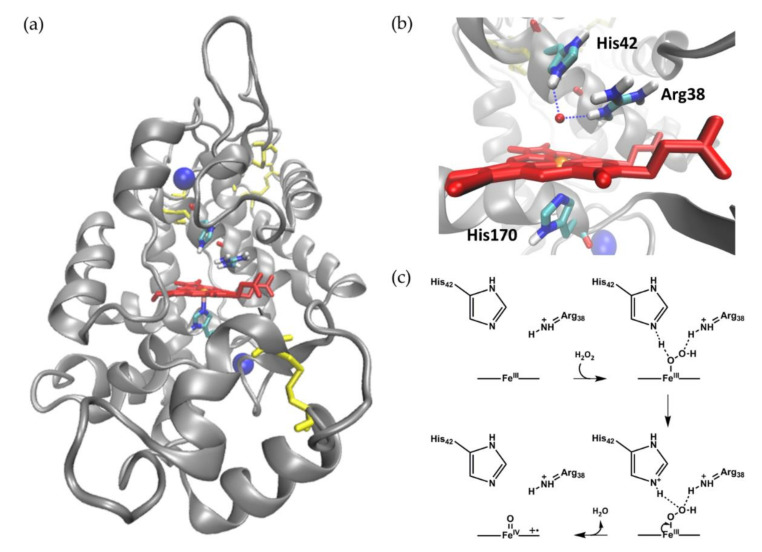
Crystal structure of HRP and mechanism for hydrogen peroxide reduction. (**a**) Overall globular structure of HRP C1A. The polypeptide chain is shown as the grey cartoon, the heme cofactor, disulfide bonds, and selected amino acids as red, yellow, and multi-colored sticks, respectively. The two calcium ions are shown as blue spheres. (**b**) Active site of HRP C1A with the heme cofactor depicted as red sticks with the iron center as orange sphere. The essential amino acids Arg38, His42, and His170 are shown as sticks colored by element. Structures were visualized using PDB 1ATJ [33] and VMD 1.9.3 [37]. From Neumann, 2019 [38]. (**c**) Proposed mechanism for hydrogen peroxide reduction by heme peroxidases and concomitant Compound I formation. Adapted with permission from Rodríguez-López et al., 2001 [34]. Copyright (2001) American Chemical Society.

Figure 1 shows the overall structure of HRP isoenzyme C1A as well as the active site arrangement which is common in all plant peroxidases. HRP C is a monomeric and mainly α-helical glycoprotein with a molecular weight of 44 kDa. Furthermore, HRP contains four disulfide bonds and two calcium ions, both of which are important for the enzyme’s structural stability [32,33]. Figure 1b shows a close-up of the active site highlighting the cofactor and three amino acids, which have been identified as highly conserved and essential for the high activity of heme peroxidases. His170 coordinates the heme iron on the proximal side and anchors it in the center of the enzyme. The sixth coordination site is the substrate binding site and vacant in the enzyme’s resting state. During catalysis, the distal amino acids Arg38 and His42 facilitate the efficient heterolytic cleavage of the bound peroxide by abstracting and donating protons and by stabilizing leaving groups, as depicted in Figure 1c [34,35]. Upon reduction of hydrogen peroxide, water is released and Compound I, a high-valent reaction intermediate with an Fe-oxoferryl center in oxidation state +IV and a porphyrin-based cationic radical, is formed. The three main reaction steps of heme peroxidase catalysis are described with Equations (2)–(4) [32,35]:

Peroxidase + H_2_O_2_ → Compound I + H_2_O, (2)

Compound I + AH_2_ → Compound II + AH▪, (3)

Compound II + AH_2_ → Peroxidase + AH▪ + H_2_O. (4)

In two consecutive one electron transfer steps, the resting state is restored from Compound I via formation of the second intermediate Compound II. The latter is structurally similar to Compound I, but lacks the porphyrin-based radical. Single-electron or hydrogen atom donors serve as electron sources and are oxidized to radicals, which in turn can form dimers or higher oligomers. 

### 2.2. Peroxidase Reactions in Electrochemical Biosensors

**Figure 2:**
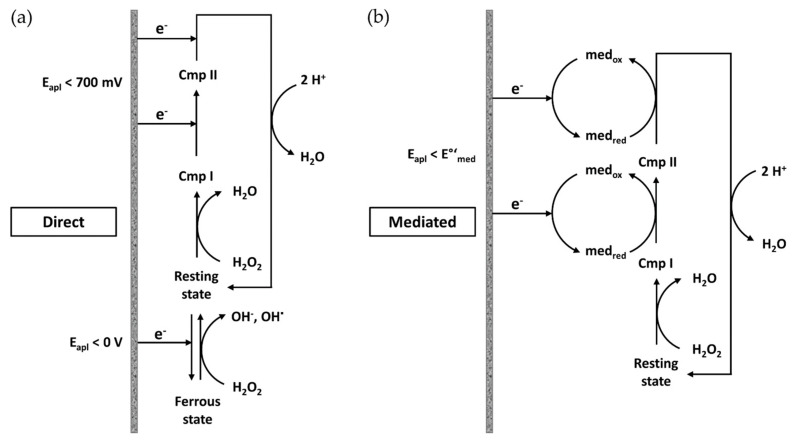
Electrochemical transduction modes of peroxidase-based biosensors. (**a**) In the direct approach, the reaction intermediates Compound I and II are directly reduced at the electrode at high potentials. The Fenton-type reaction route is initiated by conversion of the heme cofactor from the ferric (Fe^3+^) to the ferrous (Fe^2+^) state at lower potentials. (**b**) In the mediated approach, an additional compound is used to mediate electron transfer between working electrode and Compounds I and II. Peroxidase substrates can act similar to mediators when their products can be recycled at the electrode.

Figure 2 illustrates the two different measuring modes of electrochemical biosensors for the detection of hydrogen peroxide by heme peroxidases. In the direct approach, the intermediates Compound I and II formed upon reaction of the enzyme with hydrogen peroxide are both reduced at the electrode via direct electron transfer (Figure 2a). This way, the resting state is restored and a new turnover can be initiated. The reduction potentials of the redox couples Compound I/Compound II and Compound II/resting state were shown to be as high as +700 mV vs. Ag/AgCl at pH 7 [2,36]. Therefore, the electrocatalytic reduction of hydrogen peroxide by heme peroxidases can be detected at highly positive potentials.

However, often electrocatalysis is reported to occur at much more negative potentials close to the formal potential of the Fe^2+/3+^ transition. In these cases, the reaction most likely proceeds via a Fenton-like mechanism involving first the reduction of the ferric resting state by the electrode followed by a reaction of the ferrous enzyme with hydrogen peroxide to form a hydroxyl anion and a hydroxy radical (Figure 2a) [39]. Alternatively, the ferrous peroxidase could react with hydrogen peroxide to form water and Compound II which is subsequently reduced by the electrode [22]. In both cases, the reaction is initiated by generation of ferrous heme which usually requires very negative working potentials [23]. This has the disadvantage that many compounds and background reactions, especially oxygen reduction reactions, can interfere with the detection. Therefore, biosensors based on the direct reduction of Compounds I and II are preferred as they can operate at moderate potentials. The mediated approach, on the other hand, employs additional compounds that shuttle electrons between the electrode and the reaction centers of Compounds I and II (Figure 2b). Thus, they can increase the sensitivity for peroxide while the working potential of the biosensor can be tuned according to the reduction potential of the mediator. The reactivity of peroxidases with mediators thereby varies for enzymes from different sources and can be even tuned by enzyme engineering [40]. As many substrates of peroxidases, like phenolic compounds or aromatic amines, are oxidized to redox active products, also their concentration can be determined via their reduction at the working electrode following the scheme in Figure 2b [2].

### 2.3. Engineering of Heme Peroxidase Mimics

In most common heme peroxidases from plants, the heme cofactor is centered in the middle of the protein matrix and the glycosylation shell can hinder an efficient direct electron transfer to the heme active site, which may lead to a significant decrease in sensitivity or even completely prevent a direct detection of peroxides. In a number of groundbreaking studies, it was reported that the rate of direct heterogeneous electron transfer of HRP on polycrystalline gold could be increased from 1 to up to 400 s^−1^ when the deglycosylated form of the enzyme (dgHRP) was used and engineered surface cysteines and tags were employed for its immobilization [41,42]. Consequently, the sensitivity of dgHRP towards hydrogen peroxide at high potentials was reported to be more than 100 times higher compared to the glycosylated enzyme [43]. These findings demonstrated that a size reduction as well as an oriented surface immobilization of the enzyme significantly influence its electrocatalytic activity as both factors lead to a decrease of the electron transfer distance between the redox site and the electrode. 

**Figure 3:**
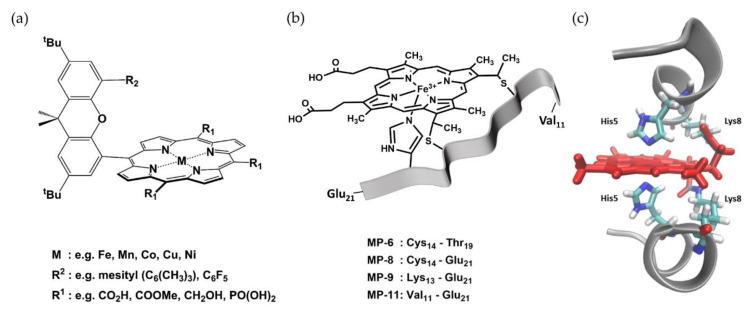
Structures of selected peroxidase mimics. (**a**) Chemical structure of a Hangman porphyrin with a xanthene linker. Various combinations of meso-substituents (R1), metal centers (M), and hanging groups (R2) have been reported. Examples for each functionality are given below. Taken from [38]. (**b**) Illustration of microperoxidases with the heme in black lines and the polypeptide chain as a grey ribbon. The respective polypeptide segments of the different microperoxidases are noted below. (**c**) Crystal structure of Co-mimochrome IV with polypeptide chains as grey cartoon, the heme cofactor, and selected amino acids as red and multi-colored sticks, respectively. PDB 1PYZ [50] visualized with VMD 1.9.3. [38].

The heme cofactor itself, the smallest catalytic unit of heme peroxidases, exhibits efficient direct electron transfer on various surfaces and its inherent peroxidatic activity has been exploited for the construction of manifold biosensors. However, compared to HRP, its enzymatic activity is around 1000 times lower due to the lack of amino acids essential for efficient catalysis [44]. The possibility to chemically modify the heme cofactor e.g., by introduction of new functional groups, led to engineering approaches for a variety of heme derivatives. Introduction of pyrrole-, thienyl-, and phenoxy-groups to the porphyrin scaffold, for instance, enabled its polymerization and formation of films with electrocatalytic activity [45,46,47,48]. Furthermore, by coupling a rigid linker to the macrocycle, functional groups were positioned in the second coordination sphere of the iron center, as shown in Figure 3a. These so-called Hangman-porphyrins constitute a group of simplified mimics of the active site of heme peroxidases. It was reported that an Fe-Hangman porphyrin bearing an acidic group in the second coordination sphere showed a three orders of magnitude higher activity towards hydrogen peroxide reduction compared to the Fe-porphyrin lacking the hanging group [49]. It was proposed that the proton abstracting/donating properties of the hanging group facilitate the heterolytic cleavage of bound hydrogen peroxide and formation of a reaction intermediate similar to Compound I of heme peroxidases. 

Additionally, various biomolecules were shown to increase the activity of the heme cofactor upon complex formation by preventing its aggregation and providing acid-base functionalities for catalysis. The interaction partners range from G-quadruplexes (DNAzymes) [51] and monoclonal antibodies (Hemoabzymes) [52] to short peptides, including amyloid β peptides involved in Alzheimer’s disease [53,54]. Presumably, the most prominent examples of peroxidase mimics though are microperoxidases. These mini-enzymes with peroxidatic activity are prepared via proteolytic digestion of cytochrome *c*, leading to formation of heme-peptide complexes with a polypeptide chain of typically six to eleven amino acids (MP-6 to MP-11), as shown in Figure 3b [55]. The recently reported fully synthetic approach for the synthesis of microperoxidases further opened up new possibilities for the design of customized heme-peptides incorporating even non-natural groups [56]. Microperoxidases were shown not only to possess a peroxidase-like activity, but also to catalyze dehalogenation reactions [57] and oxygen-transfer reactions similar to cytochrome P450 enzymes [58], making them attractive tools for the construction of biosensors. Nastri et al. also pursued an approach for the rational design of heme-peptide conjugates. By using the β-chain of human deoxyhemoglobin as the template, they designed mimochrome I, a complex composed of two helical peptide chains and a deuteroporphyrin arranged in a helix-heme-helix sandwich structure [59]. Figure 3c shows exemplarily the crystal structure of Co-mimochrome IV, one of the subsequently created mimochrome variants incorporating different metal centers and polypeptide chains [50]. The prototype Fe-mimochrome I was characterized by two symmetrical peptide chains with histidine coordinating the central metal ion on both sides, and thus did not show significant peroxidase activity. Fe-mimochrome VI on the other hand, obtained after iterative optimization, has an asymmetric structure and a vacant coordination site at the iron center [60,61]. The most recent mimochrome was reported to be highly active towards oxidation of classic peroxidase substrates as well as nitration of phenols, with the reaction proceeding via formation of Compound I [62]. 

## 3. Peroxidase Reactions on Semiconductors for Electrochemical Biosensing

### 3.1. Semiconductors as Electrode Materials for Biosensors

The use of inorganic or organic semiconductors in potentiometric sensors like field-effect transistors is well-established. These can be easily miniaturized employing microelectronics or screen-printing technologies, enabling, for example, the fabrication of integrated sensor arrays and even their deposition on flexible surfaces [63,64]. The coupling of semiconductors and illumination is widely used for photovoltaic systems with either the semiconductor itself as light-active component or in combination with an immobilized photosensitizer as a light-harvesting unit [20,65]. Photoswitches constitute another group of possible applications for semiconductors in combination with biomolecules. In enzyme photoswitches, for example, the activation of charge carriers of the semiconductor by internal or external irradiation induces redox changes in an immobilized enzyme and thereby initiates a substrate conversion [21]. The photocurrents resulting from these photo(electro)catalytic processes can then be detected in dependency of the substrate concentration. 

The comparatively low conductivity of semiconductors can result in slow direct electron transfers and high background currents. However, their conductivity can be tuned by varying the material composition e.g., by metal ion doping. In addition, a general prerequisite for the construction of an electrochemical biosensor is an appropriate surface that facilitates a productive immobilization and stabilization of the target biomolecules. Analogous to other materials, the surface of semiconductors can be designed according to the requirements given by the properties of the catalyst. Transparent conducting oxides (TCOs) form a group of optically transparent semiconductors including e.g., the widely used titanium dioxide or indium tin oxide (ITO) [66]. They provide a biocompatible matrix for catalyst immobilization that enables the combination of electrochemical and spectroscopic transmission measurements in the visible range of the spectrum. This is explicitly attractive for the analysis of surface-confined heme peroxidases or their mimics as the heme cofactor exhibits a strong absorbance in this range. The absorbance wavelength is sensitive to its redox state as well as its immediate environment, thus allowing for example the identification of reaction intermediates [22]. Additionally, the oxidation products of the peroxidase reaction are often characterized by a high fluorescence or pronounced absorbance in the visible range, thus enabling a facile spectroscopic monitoring of the heme peroxidase activity. Enzymes were adsorbed to planar or porous TCO-substrates as well as to TCO-nanomaterials [18,67,68]. For covalent coupling surfaces can be further modified with different functionalities e.g., by formation of silane- [1,69], phosphonic acid- [69,70], or aryl diazonium salt-based self-assembled monolayers (SAMs) [71,72]. The sol-gel process is a well-established procedure for the preparation of TCOs based on the hydrolysis of metal alkoxide precursors like tetramethoxysilane [73]. Sol-gel materials offer a tunable porosity and biocompatibility and are characterized by a high optical transparency as well as mechanical, chemical, and thermal stability and negligible swelling in various solvents. Moreover, refinements in the experimental conditions with respect to the used solvents, pH, as well as the temperature required for the final drying step paved the way for the addition of biomolecules during the sol or gel formation, thus enabling their encapsulation during fabrication [74]. 

Organic semiconductors, polymers like polythiophene and polypyrrole (PPy), have also been widely used for the construction of peroxidase-based biosensors. Electropolymerization often serves as a method for a highly controllable deposition of these polymers onto conductive surfaces. Given suitable conditions, the electropolymerization performed in presence of the enzyme leads to its entrapment and thus enables a one-step synthesis of electrochemical biosensors [75]. Besides their physical entrapment into electropolymerized films or the subsequent adsorption, approaches for covalent electropolymerization of biomolecules bearing polymerizable groups have been reported. These range from DNA fragments [76] to whole enzymes as GOx, where its copolymerization via pyrrole groups led to a higher activity in the film than achieved by entrapment [77]. 

### 3.2. Biosensors with Natural Heme Peroxidases

In 1989, Tatsuma et al. reported the first immobilization of HRP on tin oxide coated glass [14]. Amino groups were introduced on the semiconductor’s surface via silanization with (3-aminopropyl)triethoxysilane (APTES) followed by covalent coupling of the enzyme. The thus obtained biosensor detected hydrogen peroxide at +150 mV vs. Ag/AgCl with ferrocenecarboxylic acid as mediator. By additional coupling of GOx, the sensor was further successfully employed for the detection of glucose. Afterwards, plenty of publications on electrochemical biosensors employing peroxidases on TCO materials followed. Wu et al. published the first encapsulation of HRP in a sol-gel matrix and reported the preservation of its enzymatic activity inside the glass demonstrated by dibenzothiophene oxidation [78]. Studies for hydrogen peroxide detection via chemiluminescence [79] as well as cholesterol detection via co-entrapment of HRP and cholesterol oxidase [80] followed. Lloyd et al. transferred the sol-gel system to 96-well microplates and demonstrated the protective effect of the enzyme encapsulation at high hydrogen peroxide/HRP ratios during 3,3′,5,5′-tetramethylbenzidine oxidation [81]. However, the spectrum of application of sol-gels in biosensors was quickly expanded from only optical to electrochemical read-out. At first, most approaches focused on the deposition of sol-gels on non-transparent materials like carbon with HRP encapsulated in, adsorbed on or covered with the sol-gel matrix for fabrication of biosensors for hydrogen peroxide either in absence [82,83,84] or presence [85,86,87] of a mediator. Chen et al. additionally doped a silica based sol-gel with multi-walled carbon nanotubes, leading to a four times higher sensitivity towards hydrogen peroxide compared to the matrix lacking the nanotubes [88]. Furthermore, TCO nanomaterials like various zinc oxide nanostructures [89,90], antimony oxide bromide nanorods [91], as well as iron and cobalt oxide nanoparticles [92,93] have been employed for HRP immobilization on non-TCO materials. Additionally, titanium dioxide has been used in the form of soluble nanoparticles and nanotubes [94,95], but also as nanotube arrays directly grown on titanium foil via anodic oxidation [96,97,98,99]. Kumar et al. reported that the introduction of such a nanoporous oxide layer enabled a direct electrochemical communication between adsorbed HRP and the electrode which was not observed on titanium alone [99]. Also for a mediated approach a significant increase in the hydrogen peroxide reduction by HRP was obtained when the enzyme was immobilized in a graphite composite with mesoporous TiO_2_ rather than non-porous TiO_2_ [100]. Alternative attempts to improve this communication included the incorporation of gold nanoparticles in between the enzyme and the TCO substrate [101]. However, in the afore-mentioned systems, a direct reduction of hydrogen peroxide was only observed at negative potentials down to -0.6 V vs. Ag/AgCl, thus indicating a Fenton-type reaction at the electrode and not the electrocatalytic behavior expected for HRP. 

**Figure 4:**
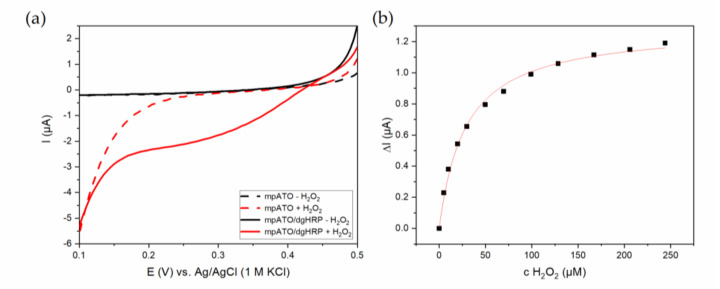
Electrocatalytic reduction of hydrogen peroxide by dgHRP adsorbed on mpATO. (**a**) Linear sweep voltammograms of bare (dashed lines) and dgHRP-modified (solid lines) mpATO before (black) and after (red) addition of 2 mM hydrogen peroxide in air-saturated 100 mM phosphate buffer, pH 7.4. Scan rate 2 mV/s, stirring speed 500 rpm. (**b**) Concentration dependent current increase of a dgHRP modified mpATO upon hydrogen peroxide addition obtained from amperometric measurements at 0.2 V vs. Ag/AgCl. Data were fit to the Michaelis–Menten equation. Adapted from [38].

In 2009, Astuti et al. reported on the direct spectroelectrochemistry of HRP and cytochrome *c* peroxidase immobilized on mesoporous TiO_2_ as well as polylysine modified mesoporous SnO_2_ employed for electrocatalytic measurements [102]. Here, the authors were able to confirm the formation of reaction intermediates Compound I and II on the electrode surface by following spectroscopic changes as well as by the high onset potentials of the cathodic reduction of hydrogen peroxide. However, they also reported that HRP showed a much less favorable heterologous electron transfer than cytochrome *c* peroxidase due to its glycosylation shell, which aside from its insulating and distance-increasing effects, could also hinder a proper access to the pores. The use of engineered HRP-variants could circumvent this problem as has initially been shown for gold electrodes [103]. Our group recently immobilized His_6_-tagged dgHRP on a mesoporous TCO electrode support and investigated its spectroelectrochemical as well as electrocatalytic properties [38]. Here, antimony tin oxide (ATO) was employed due to its previously discovered binding affinity for His_6_-tags [18,104]. A direct electronic communication of the heme center with the electrode surface was demonstrated by spectroelectrochemical measurements as well as electrocatalytic reduction of hydrogen peroxide in absence of a mediator. The larger potential window of ATO in comparison to SnO_2_ enabled the determination of the reduction onset potential. The latter was with +439 mV vs. Ag/AgCl high enough to confirm the formation of Compounds I and II and to enable hydrogen peroxide determination in aerobic conditions without interference of oxygen (Figure 4a). While the linear concentration range was comparable to that of HRP on PLL-modified mesoporous SnO_2_, the sensitivity was significantly lower, which can be attributed at least in part to the 400 mV higher working potential of our system where the Fenton-type reaction is avoided (Figure 4b, Table 1). 

**Figure 5:**
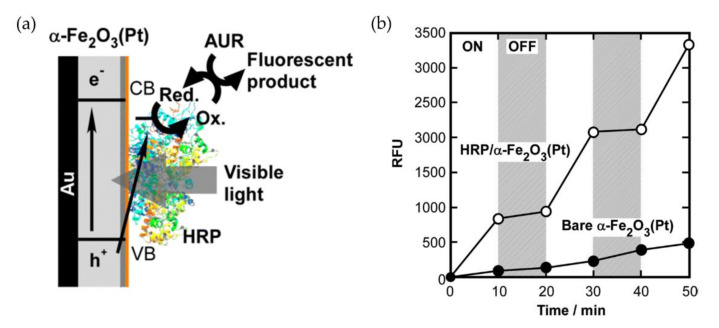
Light-driven conversion of Amplex Ultrared (AUR) by HRP on Pt-doped hematite thin films. (**a**) Schematic illustration of photoinduced enzymatic reaction by HRP adsorbed on Pt-doped α-Fe_2_O_3_ thin film. AUR is catalytically oxidized to a fluorescent product by the HRP bound to the film under visible light illumination. (**b**) Photoswitching behaviors of catalytic oxidation of 0.1 mM AUR by bare or HRP-adsorbed α-Fe_2_O_3_(Pt) under intermittent blue light irradiation (2 mW/cm^2^). Reprinted with permission from Kamada et al., 2012 [108]. Copyright (2012) American Chemical Society.

While the vast majority of peroxidase-based electrochemical biosensors were constructed for the determination of hydrogen peroxide, only a few reports on phenol detection by peroxidases on semiconductors have been published. Rosatto et al. exploited the comparatively low conductivity of silica gels for suppression of the direct reduction of hydrogen peroxide by HRP on a carbon paste electrode and thereby increased the biosensor’s sensitivity for various phenolic substrates [4]. Dai et al. on the other hand, coupled the reaction of HRP with that of tyrosinase [105]. Co-immobilization of both enzymes on mesoporous silica yielded a biosensor that exhibited a higher sensitivity for phenol than the respective monoenzyme systems and that was also applied for detection of catechol and *p*-cresol. Interestingly, the addition of hydrogen peroxide was not required in this system as it was generated in situ via the reduction of dissolved oxygen. Hydrogen peroxide is also produced via oxygen reduction by irradiated TiO_2_ and functioned as oxidant for the soybean peroxidase catalyzed oxidation of 2,4,6-trichlorophenol by a TiO_2_-soybean peroxidase composite material [27]. Here, the enzyme and the TCO material were entrapped in an UV-cured acrylic polymer matrix coated on glass. The authors reported that in presence of the enzyme less toxic intermediates are formed during degradation than by TiO_2_ alone, making the system more attractive for bioremediation applications. Kamada et al. reported an increased UV-tolerance of HRP intercalated into semiconducting titanate layers [106], a circumstance they then exploited for the photoswitched oxidation of Amplex Ultrared initiated by direct oxidation of bound HRP to Compound I upon UV-irradiation of Fe-doped titanate [107]. Subsequent oxidation of the substrate by the reaction intermediate was followed by formation of the fluorescent product. The authors demonstrated a precise control of the enzymatic activity by irradiation without the need for external or in situ produced hydrogen peroxide. Later, the group further modified this approach by immobilizing HRP on a layer of platinum doped hematite on gold or platinum supports, as shown in Figure 5. The more narrow band gap of hematite compared to titanate enabled the initiation of the enzymatic reaction by visible light irradiation [108]. Though the authors did not report on the construction of a biosensor, this approach can avoid photodeactivation of peroxidases, making it an attractive starting point for the development of various applications. 

In 1990, Wollenberger et al., reported for the first time the one-step fabrication of a hydrogen peroxide biosensor based on entrapment of HRP in a PPy matrix during electropolymerization on pyrolytic graphite and platinum [109]. Here as well, a bienzyme approach for glucose determination was established, employing a laminated GOx membrane on top of the electrode. Despite a decrease in sensitivity for H_2_O_2_ compared to directly adsorbed HRP, a significant increase in long-term stability was observed. The HRP/PPy system was shortly after transferred to tin oxide by Tatsuma et al. in 1992 who measured with 10 nM a four magnitudes lower detection limit for hydrogen peroxide [110]. In both cases, electrocatalytic reduction was observed at potentials much more positive than the Fe^2+/3+^ transition. However, the question remained, if PPy served as conducting matrix facilitating electron transfer between HRP Compounds I and II and the electrode or if this process was mediated by pyrrole dimers entrapped in the polymer matrix. Both hypotheses were found to be eligible as Tatsuma et al. demonstrated the usability of PPy as conductive material as well as the ability of pyrrole oligomers to function as mediators [110]. Later, the group also extended their system to glucose detection by incorporation of GOx during electropolymerization [111] while Yoshida et al. coupled the HRP reaction with that of glutamate oxidase for fabrication of a mediator-free glutamate sensor [112]. Further optimizations of the HRP/PPy system with respect to hydrogen peroxide detection were reported. For example, coating the HRP/PPy electrode with a film incorporating catalase as “substrate purging catalyst” increased the upper limit for hydrogen peroxide detection by two orders of magnitude [113]. Razola et al. further deposited a thin PPy layer between electrode surface (platinum or glassy carbon) and enzyme layer in order to prevent denaturation of HRP and surface blocking [114]. Indeed, the obtained sensor detected hydrogen peroxide at +150 mV vs. Ag/AgCl in a lower concentration range than the initially reported system [109], but could not reach the sensitivity of HRP/PPy on SnO_2_ [110]. Intriguingly, Razola et al. excluded the possibility of electron transfer mediation by pyrrole or its oligomers in their system and declared a direct electrocatalytic reduction of hydrogen peroxide by HRP. 

**Figure 6:**
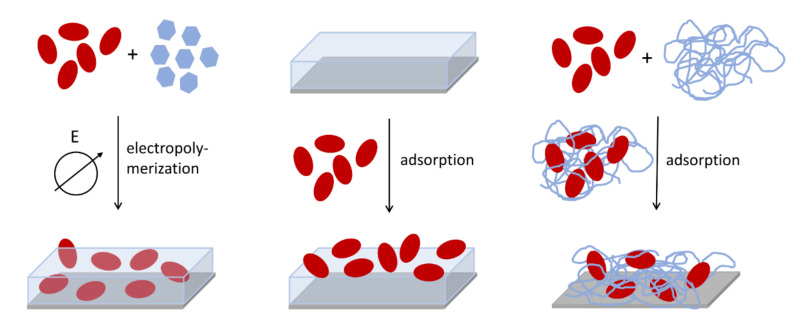
Schematic illustration of different ways of combining biomolecules and organic semiconductors for the fabrication of sensors: entrapment during electropolymerization or co-polymerization, adsorption to pre-polymerized films or adsorption of enzyme/polymer mixtures on the surface.

As alternative to entrapment of peroxidases in electropolymerized films, various approaches based on either grafting of enzyme/polymer mixtures on surfaces or adsorption of the catalyst on pre-formed polymer films have been published (Figure 6). Both procedures have the advantage that less enzyme amounts are required than for batch polymerization. But, in contrast to pyrrole, for example, polymers used for mixing with enzymes need to be soluble in aqueous solution in order to ensure that the catalyst retains its activity. Again, Tatsuma et al. were among the first to report an electrochemical biosensor based on various peroxidases mixed with the water-soluble polymer poly(3-(3’-thienyl)propanesulfonic acid [115]. The mixture was cast on SnO_2_ and electrocatalytic hydrogen peroxide reduction was observed at potentials up to 1 V vs. Ag/AgCl. By removal of thiophene monomers and oligomers after the chemical polymerization, the authors excluded participation of these species as mediators in the electron transfer and concluded that the reaction intermediates of HRP, a microbial peroxidase and lactoperoxidase received electrons directly from the polymer matrix. Moreover, using this hydrophilic polymer enabled stable measurements in the organic solvent acetonitrile [116]. For several systems based on adsorption of HRP on polymer films, aniline was the monomer of choice. In 1997, Yang et al. deposited polyaniline on platinum foil or glassy carbon via electropolymerization and subsequently adsorbed the positively charged enzyme during reduction of the polyaniline film at -0.5 V vs. SCE [117]. The thus constructed sensor detected hydrogen peroxide at moderate potential without the need for a mediator (Table 1). Hua et al. used composites of polyaniline and multiwalled carbon nanotubes for immobilizing HRP on gold and obtained a biosensor detecting hydrogen peroxide with a high sensitivity, though at negative potentials (Table 1) [118]. Bartlett et al. on the other hand,Bartlett et al. exploited the direct electrochemical communication between HRP and polyaniline for the fabrication of an enzyme switch, a so-called microelectrochemical enzyme transistor [119]. Here, HRP was adsorbed to electrodeposited polyaniline on dual carbon microband electrodes. Compounds I and II formed upon reaction of HRP with hydrogen peroxide oxidize the polyaniline matrix leading to a switch from its conducting to an insulating form which is then reversed by the potentiostat. The potential change of the polymer and the drain current served as measuring parameters for the concentration-dependent detection of hydrogen peroxide. Furthermore, cross-conjugated polymer networks from self-assembled nanoparticles were created by electropolymerization serving as conductive surface for HRP immobilization and the thus created biosensors were reported to be highly sensitive [120,121].

Some more recently published articles on peroxidase-based electrochemical biosensors reported on the combined use of organic semiconductors and innovative technology from areas like nanotechnology and sensor printing. Li et al., for instance, transferred the HRP/PPy system with an incorporated mediator coating to screen-printed carbon paste electrodes and thereby fabricated a disposable hydrogen peroxide biosensor for low sample volumes of down to 1 µl [122]. Qian et al. reported on the generation of microporous PPy films for HRP immobilization [123]. They adsorbed SiO_2_ spheres of defined size on gold surfaces and electrogenerated a PPy film on top, followed by etching of the silica template, thus generating homogeneous PPy films with a pore diameter of 180 nm. Subsequently, a mixture of HRP and chitosan was electrochemically co-deposited in the pores and hydrogen peroxide could be detected in absence of a mediator. Further approaches for preparation of micro- or nanostructured polymer films for HRP-based biosensors involved the generation of oxygen microbubbles during PPy electrogeneration on stainless steel [124], as well as the use of a nanoparticulate polyaniline derivative for electrodeposition [125]. Zhu et al., on the other hand, deposited an HRP/PPy layer on top of a layer of single wall carbon nanotubes and reported a 50 times increase in sensitivity for hydrogen peroxide compared to a system employing graphite powder (Table 1) [126]. The authors used this setup in combination with GOx for sensitive determination of glucose in serum samples. In 2007, Setti and co-workers presented an approach for combining organic electronics with enzyme immobilization by thermal inkjet technology [127]. They printed an organic conductive ink made of poly(ethylenedioxythiophene) (PEDOT) on an ITO-covered glass and subsequently printed an HRP-ink on top. Although the biosensor had to be covered in a cellulose acetate membrane in order to avoid leaching of the layers and a mediator was required for a sensitive hydrogen peroxide detection, this technology is a promising step towards completely printed peroxidase-based biosensors that can be also exploited for the fabrication of sensor arrays on various materials.

### 3.3. Heme-Peptide Complexes

Several heme proteins including hemoglobin and myoglobin were shown to exhibit a pseudo-peroxidase activity when immobilized on electrode surfaces where usually reduction of the heme iron initiates a Fenton-type reduction of hydrogen peroxide. Respective studies have also been conducted with gold nanoparticles on ITO [128] or TCO nanomaterials like ZrO_2_ nanoparticles, TiO_2_ nanotubes and nanosheets [129,130,131]. Only a few reports exist on the electrochemical properties of the initially hemoglobin-derived mimochromes, none of them analyzing the electrocatalysis of peroxidase-like reactions [61,132]. However, in 2014, Vitale et al. employed mesoporous ITO electrodes for immobilization and spectroelectrochemical analysis of Fe^III^- and Co^III^-mimochrome VI [133]. The authors observed a direct electrochemical communication of heme and electrode, thus paving the way for a potential application of engineered heme-peptide complexes as catalysts in mediator-free electrochemical biosensors.

Microperoxidases, on the other hand, were extensively used as catalysts in electrochemical biosensors, some of which also involved semiconducting electrode materials. In 1991, Tatsuma et al. seamlessly followed up on their work with HRP and were the first to immobilize a microperoxidase on a TCO for sensing of hydrogen peroxide [134]. Again, APTES-modified SnO_2_ served as electrode support onto which MP-9 was covalently attached via glutaraldehyde. The authors reported an almost ten times higher surface coverage of MP-9 compared to HRP and in contrast to the natural enzyme, electrocatalytic reduction of hydrogen peroxide by MP-9 was already observed at potentials of +300 mV vs. Ag/AgCl in absence of a mediator. Both effects were attributed to the significant size reduction of the heme catalyst. On the other hand, the sensitivity of the MP-9 modified electrodes was with 9 × 10^−4^ A cm^−2^ M^-l^, almost 50 times lower than that of HRP on SnO_2_ (Table 1). The linear range was shifted to higher concentrations (>1 µM) making this sensor attractive for the analysis of different samples. Using the same system, the authors also designed a biosensor for imidazole and derivates based on the inhibiting effect of these compounds on the direct reduction of hydrogen peroxide by MP-9 [135]. Astuti et al. too extended their spectroelectrochemical studies of HRP on poly-lysine modified mesoporous SnO_2_ to microperoxidases [136]. The authors reported a 30 times higher surface coverage of the heme-peptide than obtained with the natural enzyme with more than 90% of the molecules being electroactive. Here, the sensitivity of the MP-11 electrode towards hydrogen peroxide reduction was almost four times higher than that of HRP on SnO_2_ (Table 1). However, in contrast to HRP and the MP-9 system developed by Tatsuma et al., Astuti and co-workers proposed a Fenton-like reaction mechanism. Later, our group pursued a similar approach when we adsorbed MP-11 in mesoporous ATO electrodes modified with the positively charged binding promotor polydiallyldiammonium chloride [137]. Although we as well observed an almost ten times increase in surface coverage, the sensitivity was seven times lower compared to dgHRP on mesoporous ATO (Table 1), which at least in part can be attributed to formation of six-coordinated low-spin MP-11 as verified by resonance Raman spectra. Still, we observed formation of high-potential reaction intermediates demonstrated via hydrogen peroxide reduction at potentials below +450 mV vs. Ag/AgCl. A comparable behavior was observed for MP-11 in a three-dimensional layer-by-layer assembly of the heme-peptide and gold nanoparticles on APTES-modified ITO [138]. Tian et al. as well combined both materials and fabricated microstructured silica on an ITO surface with gold nanoparticles electrodeposited inside the cavities in order to immobilize MP-11 via covalent coupling to a mercaptobenzoic acid SAM [139]. The SiO_2_ cavities enhanced the electron transfer as well as the sensitivity for hydrogen peroxide reduction though in this system, the latter was observed only at negative potentials. Renault and co-workers performed extensive spectroelectrochemical analyses of MP-11 in porous ITO and TiO_2_ with respect to the MP-11-catalyzed electrocatalytic reduction of molecular oxygen and used it as a model compound for thorough investigations of electron and charge transfer processes in porous TCO electrodes [67,140,141,142]. Much less attention on the other hand has been directed to combinations of microperoxidases and organic semiconductors for biosensor construction. Korri-Youssoufi et al. transferred the well-established HRP/PPy-system to MP-8 and reported a calculated limit of detection for hydrogen peroxide of 3.7 nM [143].

**Figure 7:**
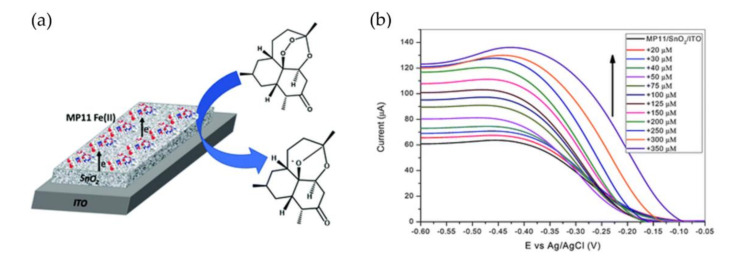
Artemisinin (ART) sensing by MP-11 on mesoporous SnO_2_. (**a**) Schematic representation of MP-11 immobilized on didodecyldimethylammonium bromide (DDAB) modified SnO_2_ film electrodes causing the electrocatalytic reduction of ART. (**b**) DPVs of the sensor after the addition of increasing ART concentrations in 10 mM NaH_2_PO_4_ pH 7 buffer at a scan rate 0.1 V s^−1^. Reprinted from Ioannidis et al., 2019 [146]. Published by the Royal Society of Chemistry.

Additionally, microperoxidase/semiconductor systems have also been used for the electrochemical detection of other compounds than hydrogen peroxide, including glucose via combination with glucose oxidase [144] or nitric oxide [145]. Recently, Ioannidis et al. presented an approach for the detection of the antimalarial drug artemisinin where MP-11 was adsorbed to a film of surfactant-modified mesoporous SnO_2_ on ITO [146]. Ferrous MP-11 catalyzes the cleavage of an organic peroxide within the artemisinin molecule, which leads to heme re-oxidation, thus invoking an enhanced electrocatalytic reduction current proportional to the concentration of the drug in solution (Figure 7).

### 3.4. Hemin and Other Fe-Porphyrins

Due to the aforementioned inherent peroxidase activity of the heme cofactor, hemin and other types of Fe-porphyrins have been widely used as catalysts for the electrochemical determination of the typical peroxidase substrates hydrogen peroxide [44,147,148] or phenolic compounds [149] as well as other substances like superoxide [150], and peroxynitrite [151]. In addition, the MP-11-based artemisinin sensor mentioned in Section 3.3 was initially developed using hemin as the peroxide reducing catalyst adsorbed on TiO_2_-modified silica [152]. The here achieved sensitivity for the target was almost two orders of magnitude higher compared to the MP-11-system. Other TCO-based electrochemical sensors with hemin involved its immobilization on SiO_2_-modified iron oxide particles [153] or mesoporous SnO_2_ on ITO grafted on a polyethylene terephthalate support, creating a flexible sensor for hydrogen peroxide [154]. The latter approach was further modified by the same group around Topoglidis using Metglas ribbons as support, thus enabling electrochemical as well as magnetoelastic sensing of hydrogen peroxide [155]. Unfortunately, the peroxide determination had to be performed at negative potentials in these systems, although some previous studies on gold and carbon have demonstrated that the direct electrocatalytic reduction of hydrogen peroxide by immobilized hemin can proceed at high potentials similar to heme peroxidases [44,148,156].

Combinations of TCOs with Fe-porphyrins have mainly been used for photocatalytic approaches. Amadelli et al. performed solution studies on the photooxidation of cyclohexane and cyclohexene by an Fe-porphyrin covalently linked to APTES-modified TiO_2_ [157]. The authors proposed that the ferrous porphyrin formed upon illumination reduces oxygen to reactive oxygen species like superoxide which then are involved in the oxidation of the organic substrates. A few years later, they presented an interesting modification to their system by employing a silane-modified Fe-porphyrin for direct covalent coupling to a TiO_2_ film on glass [158]. Spectroscopic analyses showed reduction of the iron center which could be reversed by oxygenation. Furthermore, they could demonstrate that the photooxidation of cyclohexane proceeded more efficiently and selectively in presence of the porphyrin than in its absence. The group of Meyer reported multiple studies on the photoinduced reaction of hemin on nanocrystalline TiO_2_ with organohalide pollutants like chloroform [159,160,161,162]. The authors spectroscopically followed the light-induced reduction of the iron center and reported that the ferrous catalyst was stable in the dark for days. In 2015, Gu et al. reported on the fabrication of a photoelectrochemical sensor for hydrogen peroxide employing hemin adsorbed on nanoporous NiO modified ITO [163]. Substrate determination was performed via photocurrent generation at -0.05 V vs. Ag/AgCl and the sensor was also successfully tested in real samples like milk or pharmaceutical eye drops.

**Figure 8:**
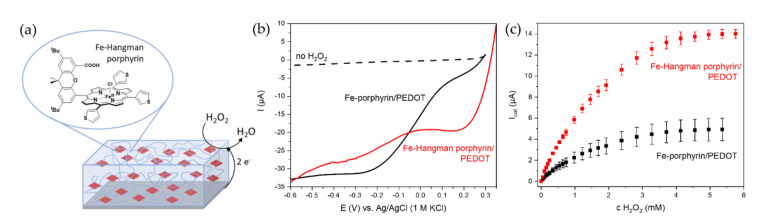
Hydrogen peroxide sensor based on electropolymerized Fe-porphyrins operating at moderate potentials in aqueous solution. (**a**) Schematic illustration of a co-polymer film of thienylated Fe-Hangman porphyrin and poly(ethylenedioxythiophene) (PEDOT) deposited on glassy carbon. (**b**) Linear sweep voltammograms of the porphyrin/PEDOT films before and after addition of 5 mM hydrogen peroxide at a scan rate of 2 mV/s. (**c**) Amperometric response of the porphyrin/PEDOT films to increasing hydrogen peroxide concentrations at 0.2 V vs. Ag/AgCl. Measurements were performed in 100 mM phosphate buffer, pH 7, at a stirring speed of 300 rpm.

The higher stability of Fe-porphyrins in organic solvents and in presence of various conducting salts compared to polypeptide catalysts offers more possibilities for its electrodeposition on a transducer surface. The catalyst can either be incorporated into a polymer matrix or it can be employed as a monomer itself. Peteu et al. entrapped hemin in a PEDOT film electropolymerized on carbon fiber electrodes and investigated its use as sensor for peroxynitrite [164]. Later, the authors employed the direct polymerization of hemin at oxidizing potentials up to 1.3 V vs. Ag/AgCl, presumably proceeding via its inherent vinyl groups, but observed a 50 times lower sensitivity for peroxynitrite [151]. Most studies on directly polymerized heme catalysts though involve Fe-porphyrins that have been chemically modified e.g., with dimethyl ester, phenyl, pyrrole, or thiophene groups with the resulting films being used for the detection of superoxide, nitrite, and nitric oxide [45,46,48,165]. However, studies about the conversion of classical peroxidase substrates by these kinds of electrocatalytic polymers are rare. Schäferling et al. observed a concentration-dependent influence of 2,4,5-trichlorophenol on the cyclic voltammograms of an Fe-porphyrin-substituted bithiophene polymer in dichloromethane but did not pursue this approach for the fabrication of a sensor [47]. Recently, our group analyzed the electrocatalytic reduction of hydrogen peroxide by thienylated Fe-porphyrins co-polymerized with EDOT on glassy carbon electrodes [166]. For the first time, we also implemented a Hangman porphyrin as catalyst and were able to demonstrate its superior catalytic behavior in terms of reduction onset and sensitivity for hydrogen peroxide at high potentials as shown in Figure 8 and Table 1.

## 4. Summary and Conclusions

The coupling of enzymes with transducer surfaces plays a key role in the fabrication of biosensors. Expanding the spectrum of both, the electrode material as well as the biocatalyst, also extends the range of opportunities for adapting the system to the specific requirements of its application. Table 1 summarizes parameters of some of the afore-mentioned systems designed for hydrogen peroxide sensing based on the natural enzyme HRP, microperoxidases as well as hemin and other Fe-porphyrins coupled to semiconductor materials. 

On average, the systems did not outperform those employing classic electrode materials. On the contrary, most sensors operated at higher concentration ranges with sensitivities considerably lower than e.g., HRP on graphite [167,168,169] or dgHRP on polycrystalline gold [103]. High charging currents as well as a pronounced direct reduction of hydrogen peroxide by the semiconductor matrix impede the measurements. Furthermore, it must be considered that the three-dimensional surface architecture of many TCO- and polymer-based systems is often not considered for current density calculations, thus resulting in higher sensitivities when the geometrical area is used instead. For several systems employing heme peroxidase mimics, a better sensitivity towards reduction of hydrogen peroxide compared to HRP was obtained as shown in Table 1. However, only a few operate at similarly high potentials, thus truly mimicking the enzymatic reaction via Compounds I and II. Furthermore, the mimics often exhibit higher apparent K_m_ values, reflecting in part a lower affinity to the substrate, resulting in higher linear ranges of the sensors. Though semiconductor materials might not be a lucrative alternative in terms of sensor performance, they in return offer new possibilities for the fabrication as well as read-out of peroxidase-based sensors. Transparent materials enable more facile spectroscopic analyses of the immobilized catalyst in the visible range of the spectrum, and thus an easier control of its active state. Immobilization on TCOs further enables the photoinduced initiation of the peroxidase reaction cycle either via direct oxidation of the catalyst or via in situ generation of hydrogen peroxide by TCOs as well as a photo-enhanced detection of hydrogen peroxide. 

Especially organic semiconductors bear a high potential for novel ways of biosensor construction. Conducting polymers together with enzymes form bioink formulations and various printing techniques enable their deposition in microstructured format on materials that can be transparent, flexible, or magnetoelastic, thus further extending their field of application [170]. Here, the replacement of natural enzymes by engineered nature-derived or fully synthetic mimics also offers new ways for the facile integration of the catalysts which has been already accomplished for various peroxidase mimics. In this context, the de novo design of peroxidase mimics with integrated functional groups using techniques like solid-phase peptide synthesis or complex organic chemistry is especially attractive. This way, catalysts optimized with respect to their immobilization and/or reactivity can be created as has been aspired e.g., for fully synthetic microperoxidases, the four-helix bundle heme protein MP-3 [171] or Hangman porphyrins. All these heme peroxidase mimics have a great potential for future application in sensors which can just be expanded by their combination with semiconductor materials in organic smart devices.

## Figures and Tables

**Table 1:** Performance of selected sensors for hydrogen peroxide based on natural peroxidases or peroxidase mimics immobilized on semiconductors.

Electrode Setup	E_appl_ (V)	Measuring Conditions	LR (µM)	Sensitivity (mA M^−1^ cm^−2^)	Reference
**A: HRP**					
HRP/APTES/SnO_2_	0.15	pH 5.9, Med.	0.01–1	50	[14,134]
HRP/PLL/mpSnO_2_	−0.2	pH 8	1–20	1070	[102]
dgHRP-His_6_/mpATO	0.2	pH 7.4	5–20	73	[38]
Nafion/HRP/ZnO/ITO	−0.2	pH 7.4	500–9000	7.45	[90]
HRP/Chi-AOB/GC	−0.11	pH 7	1–121	1.44	[91]
HRP-ZnO-chitosan/GC	−0.2 ^1^	pH 7, Med.	10–1800	n.d.	[89]
HRP/APTMS/npTiO_2_	<−0.3 *^1^	pH 7	100–1,500	2864 *	[99]
Nafion/HRP-TiO2/Gr/Au	−0.3 ^1^	pH 7, Med.	< 400	1090	[100]
HRP/SnO_2_/GC	−0.3 ^1^	pH 6	10–250	≈215 *	[84]
TiO_2_/HRP/GC	−0.15 ^1^	pH 7, Med.	80–560	488	[87]
HRP in PPy/pyrographite	0.01 ^1^	pH 7	50–1750	0.024 *	[109]
HRP in PPy/SnO_2_	0.15	pH 6.4/7.4	0.01–10	n.d.	[110]
HRP in PPy/SWCNT/Au	−0.1	pH 6.8	0.5–1000	430	[126]
HRP in PPy/SPCP	−0.3	pH 7, Med.	100–2000	33.2	[122]
HRP/PANI/Pt	0.2 ^1^	pH 6.8	1–8 *	n.d.	[117]
HRP/PANI/MWCNT/Au	−0.35	pH 7	86–10,000	194.9	[118]
HRP+polythiophene/SnO_2_	0.4	-	0.05–0.5	n.d.	[115]
HRP/PEDOT-PSS/ITO	−0.1 ^1^	pH 6.5, Med.	<1000	0.54	[127]
**B: Microperoxidases**					
MP-9/APTES/SnO_2_	0.15	pH 7.4	> 1	0.9	[134]
MP-11/PLL/mpSnO_2_	−0.2	pH 8	0.05–30	4300	[136]
MP-11/PDADMAC/mpATO	0	pH 8	10–750	10.6	[137]
[MP-11/PEI]_2_/ITO	0 ^1^	pH 6.3	25–125	2.14	[144]
[MP-11/AuNP]_5_/ITO	0 ^1^	pH 7.3	100–1000 *	92	[138]
MP-11/npSiO_2_-Au/ITO	−0.3	pH 7	2–600	1075 *	[139]
MP-8 in Ppy/GC	−0.1 ^1^	pH 7.4	1–9 *	-	[143]
**C: Fe-porphyrins**					
Fe_3_O_4_-SiO_2_-Hemin/GC	−0.4 ^1^	pH 7	1–160	1662 *	[153]
Hemin/SnO_2_/ITO-PET	−0.3	pH 7	1.5–90	n.d.	[154]
Hemin/SnO_2_-metglas	−0.4	pH 7	2–90	3191 *	[155]
Hemin/npNiO/ITO	−0.05	pH 7	0.5–500	n.d.	[163]
Fe-porphyrin-PEDOT/GC	0.2	pH 7	50–550	35.2	[166]
Fe-Hangman-PEDOT/GC	0.2	pH 7	50–1000	86.6	[166]

Note: Potentials refer to Ag/AgCl, those marked with ^1^ refer to SCE. * Values have been estimated by the authors of this review. LR-linear range, Med.–Mediator, n.d. – not determined.
